# P-2244. Prevalence of Antifungal Allergies in the General Population

**DOI:** 10.1093/ofid/ofae631.2397

**Published:** 2025-01-29

**Authors:** Meron Shiferaw, Monica Donnelley, George R Thompson

**Affiliations:** University of California, Davis, Sacramento, California; University of California Davis Medical Center, Sacramento, California; University of California Davis Medical Center, Sacramento, California

## Abstract

**Background:**

The use of antifungal agents continues to increase yearly and coincides with the growing immunocompromised patient population. Therapy may be prescribed as prophylaxis or treatment in fungal disease care. The emergence of novel fungal pathogens, antifungal resistant species, and infection control concerns pose challenges to antifungal agents' use. However patient tolerability remains a significant issue and documented allergic reactions to antifungal agents have not previously been reported.

Primary Outcome

**Figure:**
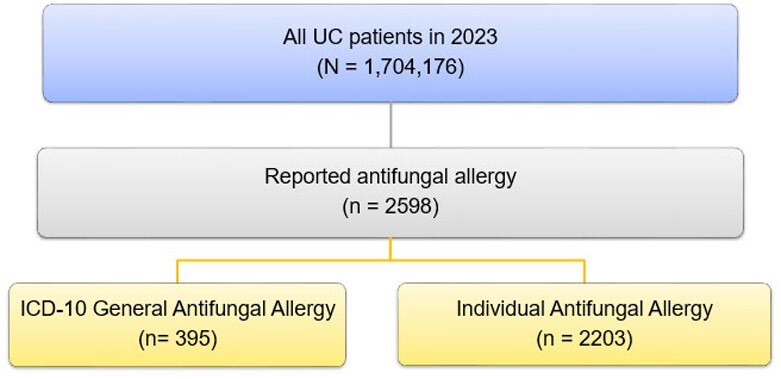

1.7 million patients assessed for reported antifungal allergy

**Methods:**

A multi-site, population health, retrospective cohort review was conducted including all healthcare sites within the University of California system. All inpatient and outpatients with an antifungal allergy reported in 2023 were included. Specifically, patients reporting an allergy to a systemic antifungal agent or with a general antifungal allergy ICD-10 T36.7. The following systemic antifungals were included: fluconazole, voriconazole, isavuconazole, posaconazole, itraconazole, amphotericin B formulations, micafungin, caspofungin, anidulafungin, and/or flucytosine. The primary outcome of this study was the rate of reported antifungal allergy among a general population. Secondary outcome, the incidence of antifungal allergies among patients with documented administration of antifungal agent.

Baseline Characteristics

**Figure:**
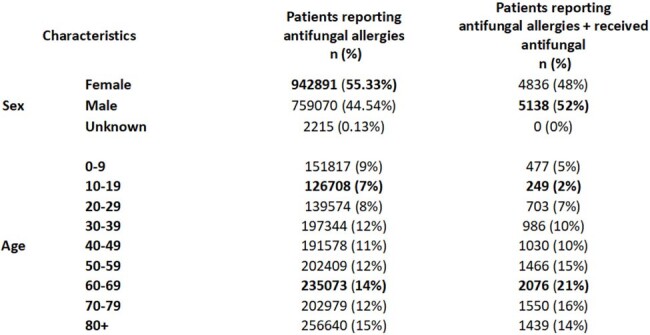

**Results:**

During the study period 1,704,176 patients were screened for reported antifungal allergy, and 2598 patients exhibited a documented antifungal allergy. An allergy to a specific antifungal agent was reported in 2203 patients, while 395 patients reported an unspecified antifungal allergy. From greatest to least reported: fluconazole 1,591 (0.093%), general antifungal allergy 395 (0.023%), triazole 361 (0.021%), itraconazole 145 (0.009%), amphotericin 110 (0.006%), flucytosine less than 10 (0.001%) (Table 1). Secondary outcome 9,976 patients received an antifungal agent and 496 reported an antifungal allergy.

Table 1

**Figure:**
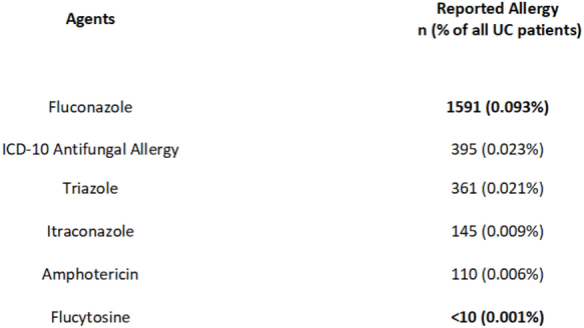

**Conclusion:**

This is the first study to report antifungal allergy rates in the general population. It assessed over 1.7 million patients and found the allergy rate to be low at 0.15%. Future work evaluating the utility of skin testing, cross-allergenicity, and desensitization strategies should be performed.

Secondary Outcome

**Figure:**
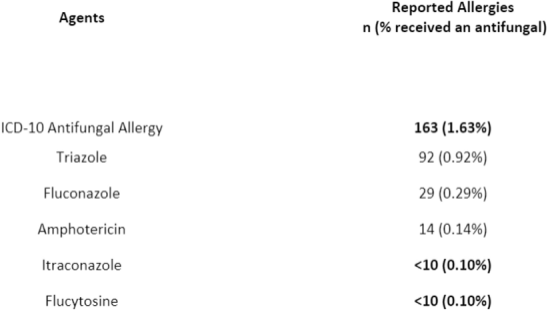

**Disclosures:**

George R. Thompson, III, MD, Astellas: Advisor/Consultant|Cidara: Advisor/Consultant|Cidara: Grant/Research Support|F2G: Advisor/Consultant|F2G: Grant/Research Support|Melinta: Advisor/Consultant|Melinta: Grant/Research Support|Mundipharma: Advisor/Consultant|Mundipharma: Grant/Research Support|Pfizer: Advisor/Consultant

